# Morphological Characterization and Molecular Phylogenetic Analysis of *Kudoa iwatai* from Large Yellow Croaker (*Larimichthys crocea*) as a New Host in China

**DOI:** 10.3390/ani12091145

**Published:** 2022-04-29

**Authors:** Xiao-Bing Li, Jie He, Rong-Rong Ma, Fu-Ying Sun, Wen-Xin Wu, Hua-Ming Luo, Lu-Huai Bai, Dong Qian

**Affiliations:** 1 College of Marine Sciences, Ningbo University, Ningbo 315823, China; 1911091081@nbu.edu.cn (X.-B.L.); marongrong@nbu.edu.cn (R.-R.M.); 17759735922@163.com (F.-Y.S.); w1778422694@163.com (W.-X.W.); b269682771@163.com (L.-H.B.); 2 Taizhou Extention Station for Fishery Technique, Taizhou 315800, China; hejie1998@sina.com (J.H.); 3634004474@163.com (H.-M.L.)

**Keywords:** myxosporea, *Kudoa iwatai*, *Larimichthys crocea*, phylogenetic analysis, identification

## Abstract

**Simple Summary:**

*Larimichthys crocea* is the most important economic marine cultured fish in China. *Kudoa* parasites are critical pathogens that infect a wide range of marine and freshwater fish. Compared to the hundred marine *Kudoa* species recognized in wild and cultured fish worldwide, records of *Kudoa* are relatively few in China. In this report, large yellow croakers were found to be infected with *Kudoa* for the first time in China. Morphological observations and molecular techniques were combined for the final identification of *Kudoa iwatai*. Additionally, the morphological characterization and phylogenetic status of *Kudoa iwatai* have been described in detail. This study enriches knowledge about *Kudoa iwatai* and provides a direction for early disease prevention and monitoring of large yellow croakers.

**Abstract:**

*Kudoa* (Myxosporea: Multivalvulida) parasites are critical pathogens in marine and freshwater fish associated with significant economic losses and reduced market prices caused by post-mortem myoliquefaction or numerous cysts on muscles. In the present study, large yellow croakers infected by *Kudoa* were found during fish disease surveillance in China in November 2020 and used for morphological observation and characterization using light DIC microscopy and electron microscopy. Numerous creamy-white oval plasmodia were observed in muscles and on the surface of brain cartilage, gill arches, and serosal surfaces. The spores were considerably longer and thicker than previously reported *Kudoa*, with protruding polar filaments (PFs) in the mature spores, fingertip-shaped apical projections (APs), and polar capsules. Phylogenetic analyses with SSU rDNA, LSU rDNA, and mitochondrial DNA showed that the *Kudoa*-infected sample (LcK-2020) had the highest similarity to *Kudoa iwatai* reported in Japan. Based on the morphological characterization and phylogenetic analysis, it could be concluded that the sample LcK-2020 was infected by *Kudoa iwatai*, which would be the first report of *Kudoa iwatai* infection in large yellow croaker in China.

## 1. Introduction

The large yellow croaker, *Larimichthys crocea*, is an economically important marine cultured fish, mainly distributed along the southeast coast of China [1]. With the establishment of artificial breeding and intensive cage culture, the large yellow croaker industry has developed rapidly in the past decade (2010–2020), with a total production of 225,549 tons per year in China, mainly in Zhejiang, Fujian, and Guangdong provinces [2] (data from China Fishery Statistical Yearbook). Due to the rapid increase in intensive mariculture and the continuous deterioration of water quality, cage-cultured large yellow croakers often suffer from severe diseases caused by parasites, bacteria, and viruses [3]. The white spot disease caused by *Cryptocaryon irritans* [4] was considered the most critical parasite infection in large yellow croaker cultures, responsible for severe economic losses. *Neobenedenia melleni* [5,6] and *Trichodina* (unpublished lab data) infections are commonly reported in cultured farms in Zhejiang and Fujian provinces every summer. In recent years, myxosporea have been reported as a common parasite in both fresh and saltwater fish, such as channel catfish (*Ictalurus punctatus*), Atlantic salmon (*Salmo salar*), and crucian carp (*Carassius auratus*) [7,8]. In addition, a few other myxosporea infections were recorded in large yellow croakers, including the newly reported myxosporean parasite *Sinuolinea* sp. [9].

The myxozoans of the genus *Kudoa* belong to the family Kudoidae (Myxosporea: Multivalvulida) and contain four or more spore valves (SVs) and polar capsules (PCs). *Kudoa*-infected fish are often accompanied by numerous white round oval pseudocysts scattered throughout muscles, often associated with post-mortem myoliquefaction due to the release of proteolytic enzymes in the fibers, also known as ‘milk flesh’, ‘soft flesh’, or ‘jelly flesh’, causing economic losses in aquaculture [10,11,12,13]. Thus far, more than 100 *Kudoa* species have been recognized in a wide range of marine wild and cultured fish worldwide [14]—mainly concentrated in the northwest Pacific and eastern Atlantic [15]. The records of *Kudoa* infections were relatively less common in China before 2008, but numbers of *Kudoa* have increased in the last decade, with new or known species reported in marine Perciformes and Mugiliformes. Here, the authors report for the first time the parasitic disease of large yellow croaker infected by *Kudoa*, identified via morphological characterization and phylogenetic analysis.

## 2. Materials and Methods

### 2.1. Sample Collection and Procession

Large yellow croaker samples with suspicious *Kudoa* infections were collected from marine cages during regular disease surveillance in Zhejiang province, East China, in November 2020, and brought to the laboratory in sealed incubators filled with crushed ice. The samples, named LcK-2020, with average specimen weights ranging from 0.5 kg to 1 kg, were necropsied within 2 h. Some white plasmodia were observed on the base of the anal fins and the dorsal fins of the fish. The tissues and organs were sampled with disposal inoculation loops for bacteria isolation with nutrient agar (NA) and thiosulfate–citrate–bile salts–sucrose agar (TCBS) under an aseptic cabinet. Plates were cultured at 28 °C for 24–48 h for bacterial observation. No predominant bacterial colonies were found in NA or TCBS agar.

Muscles, gills, and brains obviously infected with plasmodia were cut off and used for observation under the dissecting microscope. Plasmodia-infected tissues were dipped in 95% alcohol for genome extraction and fixed with 10% neutral formalin and 2.5% glutaraldehyde for histopathological and ultrastructural observation.

### 2.2. Morphological and Histological Examination

Fresh plasmodia were excised from small muscle fragments with ophthalmic scissors and squashed on a glass slide with a few drops of phosphate-buffered saline (PBS) for observation by light DIC microscopy. The dimensions of 100 mature spores were photographed and measured randomly with an ocular meter (Nikon; Corporation TOKYO; Japan), including thickness (T), sutural thickness (ST), width (W), length (L), polar filament (PF), polar capsule length (PCL), and polar capsule width (PCW). Mean values and standard deviations were calculated using the software NIC-Elements D 4.40.00 [16,17].

Parasitized tissue was extracted and placed in 10% neutral-buffered formalin for 24 h, dehydrated through a graded ethanol series, cleared in xylene, and embedded in paraffin. Histological sections 5 µm thick were prepared and stained with hematoxylin and eosin (HE) following a standard protocol and examined using light microscopy [17].

### 2.3. Ultrastructure Observation

*Kudoa* plasmodia from infected fish muscle were fixed with 2.5% glutaraldehyde in 0.2 M sodium cacodylate and post-fixed with 1% osmium tetroxide in 0.2 M sodium cacodylate. For scanning electron microscopy, part of the fixed spores was dehydrated through an ethanol series. Membranes were dried, mounted on stubs, sputter-coated with gold, and viewed with a Hitachi 3400 scanning electron microscope at 30 KV. Sections were stained in uranyl acetate and lead citrate for transmission electron microscopy and examined with a Hitachi 7700 transmission electron microscope at 120 KV [18].

### 2.4. Molecular and Phylogenetic Analysis

*Kudoa* plasmodia collected from infected muscle tissue were extracted for genomic DNA using a Tissue DNA Kit (Omega, Norcross, GA, USA), following the manufacturer’s guidelines, and qualified with a Thermo Fisher Scientific Nanodrop. The small subunit rRNA (SSU) and large subunit rRNA (LSU) of *Kudoa* plasmodia were amplified with primers Ksp18S, Kt28S1, and KI28S, respectively (Table 1). Furthermore, the cytochrome c oxidase subunit (*cox-1*) and the large subunit rRNA gene (*rnl*) of *Kudoa* mitochondria were amplified with primers of *cox-1* and *rnl*, respectively (Table 1). All PCR products were purified with a Gel Extraction Kit, cloned into a plasmid vector (pMD-19T), transformed into chemically competent *Escherichia coli* DH5α cells, and plated on LB–Amp medium overnight. Positive clones were selected for plasmid DNA extraction and amplification with universal M13 primers followed by sequencing to ensure connection accuracy.

The amplified sequences were submitted to the Basic Local Alignment Search Tool (BLAST) on the National Center for Biotechnology Information (NCBI) website to identify nucleotide sequences with high similarity. The *Kudoa* sequences of sample LcK-2020 and the sequences of *Kudoa iwatai* and other *Kudoa* species from Asian–Pacific regions were selected from the GenBank database and imported into the Mega X software and were aligned using the Align X multiple alignment program.

A phylogenetic tree was constructed with the neighbor-joining (NJ) method by applying 1000 bootstrap replicates with default settings. *Unicapsula* sp. of the family Trilosporidae, another genus belonging to the same order Multivalvulida, was used as an outgroup to construct NJ phylogenetic trees (GenBank accession nos. AY302725 and AY302727).

## 3. Results

### 3.1. Morphological and Histopathology Examination

**Figure 1:**
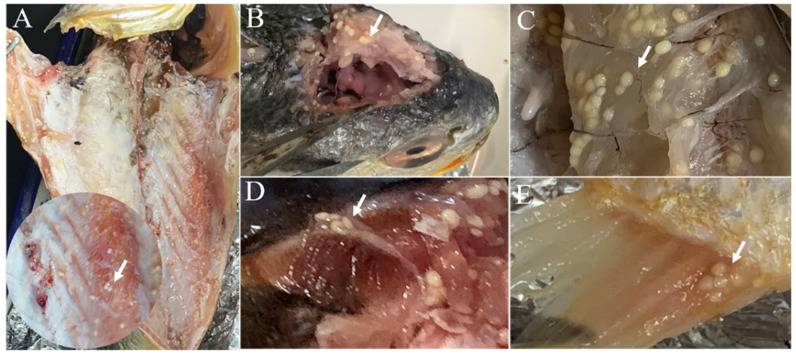
Main tissue and organs of large yellow croaker, *Larimichthys crocea*, with *Kudoa* infection. (**A**) Muscle with myoliquefaction. (**B**) *Kudoa* cysts on brain cartilage. (**C**) *Kudoa* cysts on dorsal muscle. (**D**) *Kudoa* cysts on gill arch. (**E**) *Kudoa* cyst on anal fin. White arrows: *Kudoa* cysts.

Creamy-white oval plasmodia were displayed on the base of the anal and dorsal fins of *Kudoa*-infected large yellow croakers, but no obvious lesions were found on the skin surface near parasitic sites. Numerous white cysts were observed in the muscle, with post-mortem myoliquefaction caused by the parasites. Similar cysts were also found on the surface of brain cartilage, gill arches, and serosal surfaces (Figure 1). The size of cysts in the skeletal muscle ranged from 0.1 to 0.3 cm (*n* = 100).

**Figure 2:**
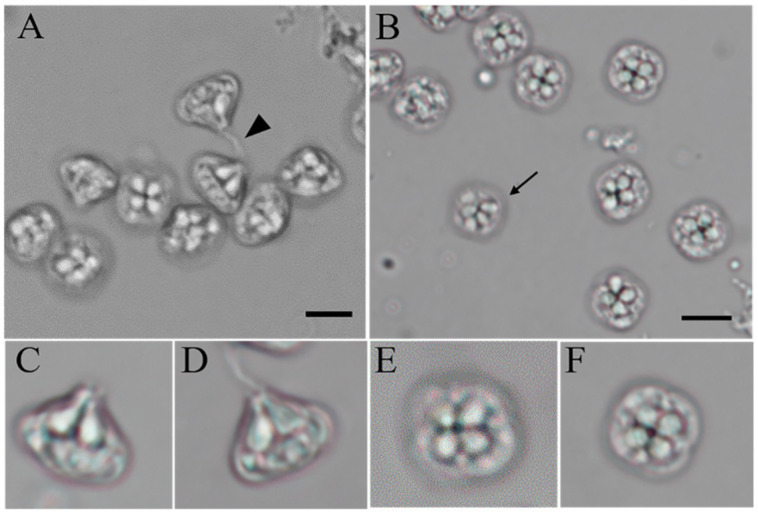
Light DIC microscopy of mature spores of *Kudoa* from large yellow croaker. (**A**,**B**) Spores of *Kudoa*. Note the protruding polar filament (black arrowhead) and aberrant spores with five polar capsules (black arrow). (**C**,**D**) Apical view of a single spore. (**E**,**F**) Lateral view of a single spore. All photographs are the same magnification. Scale bar = 5 μm.

**Figure 3:**
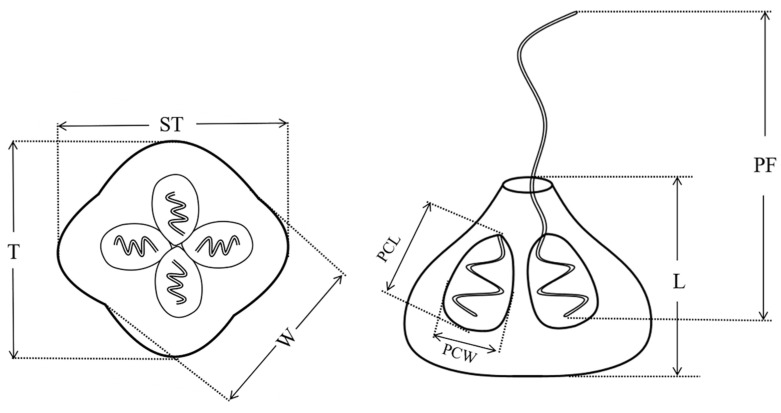
Line drawing of *Kudoa* in apical view (**left**) and lateral view (**right**) from large yellow croaker. T, thickness; ST, sutural thickness; L, length; PCL, polar capsule length; PCW, polar capsule width; PF, polar filament. Scale bar = 10 μm.

Mature quadrate spores were observed with four equal spore valves, each containing one polar capsule with a rounded peripheral edge in the apical view. The spores were garlic-shaped, and distinct apical projections on shell valves were inconspicuous. The pyriform polar capsules were equal in size, and protruding polar filaments were found on mature spores in the lateral view (Figure 2 and Figure 3). In the dimensional measurements, the average ± standard deviation (range) of spores was 8.68 ± 0.79 μm in width, 9.38 ± 0.81 μm in thickness, 10.01 ± 0.65 μm in sutural thickness, 8.15 ± 0.6 μm in length, 10.09 ± 1.03 μm polar filament length, 3.76 ± 0.65 μm polar capsule length, and 2.37 ± 0.53 μm polar capsule width. The regular morphology of the spores was measured with a microscopic view. The measurements of the *K. iwatai* from different hosts and similar species reported by other researchers are listed in Table 2. A few numbers of aberrant spores with five polar capsules were observed (Figure 2).

**Figure 4:**
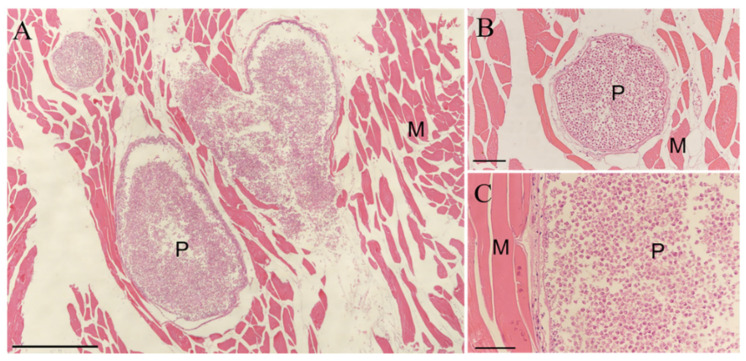
Histological analysis of muscle in large yellow croaker positively infected with *Kudoa.* H&E staining: (**A**) Scale bar = 500 μm. (**B**) Scale bar = 100 μm. (**C**) Scale bar = 50 μm. P, parasite; M, muscle.

Histological observation of infected fibrous tissue revealed that the round or oval cysts contained numerous spores delineated by thin layers of myofibers (Figure 4).

### 3.2. Ultrastructure Observation

**Figure 5:**
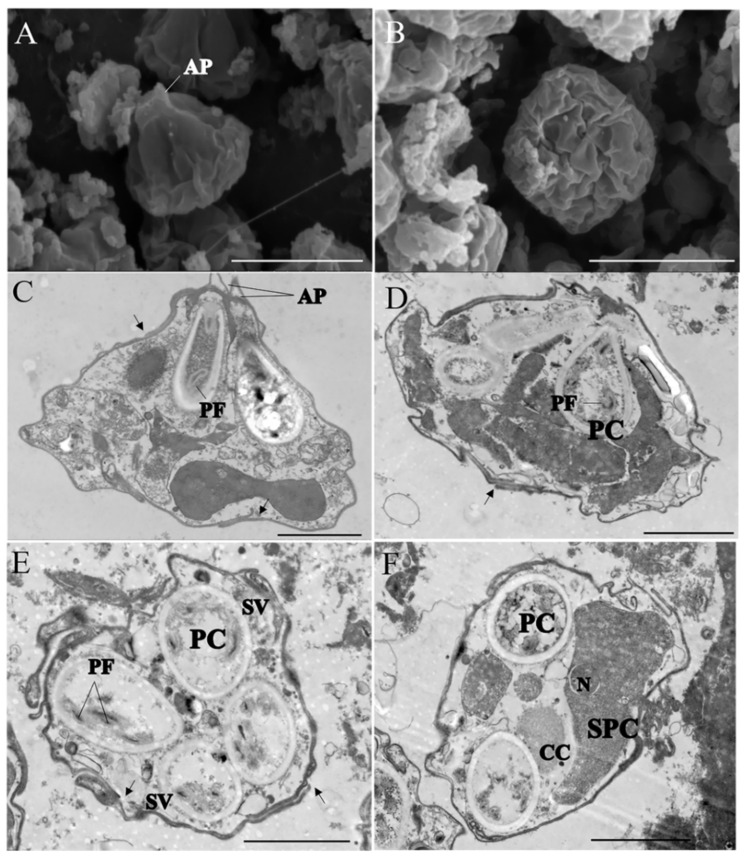
Electron microscopy of *Kudoa*. (**A**,**B**) SEM of *K. iwatai* spores. (**A**) Lateral view of *K. iwatai* by scanning electron microscopy. (**B**) Apical view of *K. iwatai* by scanning electron microscopy. Scale bar = 5 μm. (**C**–**F**) TEM of *K. iwatai*. (**C**) Ultrathin section of spore showing apical projection (AP), polar capsule (PC), and polar filament (PF). (**D**) Ultrathin section of spores showing details of desmosome-like valve junction at sutures. Note the extensive overlap of valves at sutures (black arrows). (**E**) Ultrathin section of spore showing four polar capsules (PCs), shell valves (SVs), and polar filament coils (PFs). (**F**) Ultrathin section of spore showing a capsulogenic cell (CC) with polar capsule and a sporoplasmic cell (SPC) with a nucleus (N). Scale bar = 2 μm.

The observations with both SEM and TEM revealed that the *Kudoa* spores (sample LcK-2020) were composed of four shell valves of equal size, each containing one pyriform polar capsule with 2–3 coiled polar filaments, which could be found from the lateral view of spores. Fine suture lines between each shell valve could be observed distinctly, and the oblique section of the spore showed desmosome-like valve junctions at the sutures where the valves overlapped and extended. Fingertip-shaped apical projections (APs) in some spores could be clearly observed with TEM, but APs showed square button-shaped protrusions indistinctly with SEM. The spores comprised a sporoplasmic cell (SpC) with one nucleus (N) and a capsulogenic cell with a coiled polar filament in the polar capsule (Figure 5).

### 3.3. Phylogenetic Analysis of the SSU and LSU

The sequences of small-subunit rRNA (SSU) and large-subunit rRNA (LSU) were amplified using primers of Ksp18S-F/Ksp18S-R, Kt28S1-F/Ksp28S-R, and KI-F/KI-R. The PCR products with a length of 1321 bp (SSU), 1520 bp (LSU), and 574 bp (LSU) were used for sequencing and sent to GenBank (GenBank accession numbers MW898141, MW898142, and MW898147), and followed similarity identification by the Basic Local Alignment Search Tool (BLAST) on the NCBI website (Figure S1, Supplementary File). The LcK-2020 amplified SSU rDNA sequence (MW898141) showed 99.77% identity with three *K. iwatai* isolates (LC066366, AB553294, AY641571) from *Platycephalus sp.*, *Lateolabrax japonicus*, and *Pagrus major* from Japan, and 99.54% identity with two *K. iwatai* isolates (AY514038, AY514039) from *Sparus aurata* and *Siganus rivulatus* from Israel. The LcK-2020 amplified LSU sequence (MW89814) showed 99.86% identity with two *K. iwatai* isolates (AB553303, AB638617) from *Acanthopagrus schlegelii* and *Platycephalus* sp. from Japan, and 99.75% identity with three *K. iwatai* isolates (LC066366, AB553301, AB693041) from *Platycephalus* sp. and *Lateolabrax japonicus* from Japan.

Furthermore, sequences of LcK-2020 showed high similarities to *K. lutjanus* from *Acanthopagrus latus* in China, with 99.31% and 96.41% identity to SSU (LC493818) and LSU (LC493819), respectively. LcK-2020 also showed high similarities to *K. bora* from *Osteomugil perusii* in China, with 98.77% and 94.39% identity to SSU (LC493813) and LSU (LC493814), respectively.

**Figure 6:**
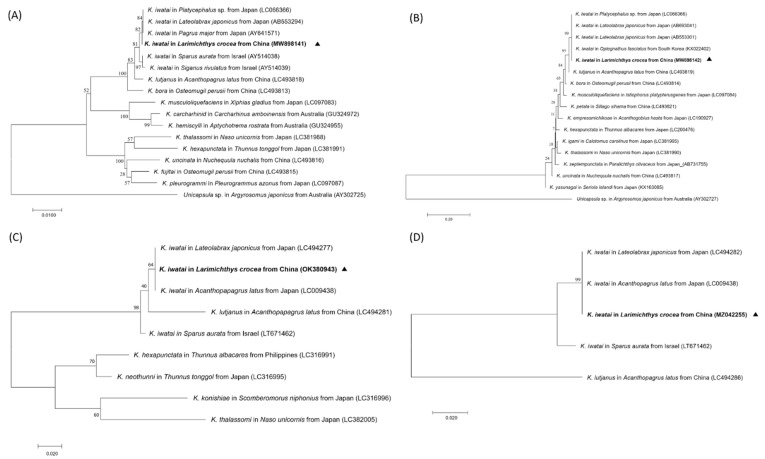
Phylogenetic trees of *Kudoa*. (**A**) Neighbor-joining (NJ) phylogenetic trees based on the SSU sequence dataset. (**B**) Neighbor-joining phylogenetic trees based on the LSU sequence dataset. (**C**) Neighbor-joining phylogenetic trees based on sequences of mitochondria of the cox-1 gene. (**D**) Neighbor-joining phylogenetic trees based on sequences of mitochondria of the rnl gene. The Kudoa species name is followed by the isolated host and isolated source and the GenBank accession number.

Phylogenetic trees were constructed for both SSU rDNA (Figure 6A) and LSU rDNA (Figure 6B), showing that LcK-2020 *Kudoa* clustered with *K. iwatai* from Japan with strong bootstrap support and a closer relationship with *K. iwatai* from Japan than *K. iwatai* from Israel or Korea. The phylogenetic tree constructed with SSU and LSU exhibited multiple representative isolates of *K. iwatai* and other closed species, such as *K. lutjanus*, *K. bora*, etc., from different host fish and geographically neighboring regions, which could be used to reveal intraspecific and interspecific genetic variation.

### 3.4. Phylogenetic Analysis of the Mitochondrial DNA Genes (cox-1 and rnl)

The *Cox-1* gene and the *rnl* gene of LcK-2020, with lengths of 458 bp and 384 bp, respectively, were amplified and sequenced (GenBank accession numbers OK380943 and MZ042255). The LcK-2020 *cox-1* sequence showed 99.38% and 99.23% identity with *K. iwatai* from *Lateolabrax japonicus* (LC494277) and *Acanthopagrus latus* from Japan (LC009438); 97.16% identity with *K. iwatai* from *Sparus aurata* from Israel (LT671462); and 91.93% identity with *K. lutjanus* from *Acanthopagrus latus* from the South China Sea (LC494281). For the other *Kudoa* species, partial multiple sequence alignment revealed that the current *K. iwatai* in *Larimichthys crocea* (OK380943) differed from *K. iwatai* from *Sparus aurata* from Israel (LT671462) with respect to three bases and differed from *K. lutjanus* in *Acanthopagrus latus* from China (LC494281) with respect to 13 bases (Figure S2, Supplementary File). The *rnl* gene sequence of the *Kudoa* sample LcK-2020 showed 100% identity with *K. iwatai* in *Lateolabrax japonicus* (LC494282) and *Acanthopagrus latus* (LC009438) from Japan. However, the *rnl* gene sequence showed 97.4% identity to *K. iwatai* from *Sparus aurata* from Israel (LT671462), with a 10-nucleotide variation (375/385).

Interestingly, the phylogenetic tree with the *cox-1* gene showed that *Kudoa* from LcK-2020 had a lower identity match with *K. lutjanus* in *Acanthopagrus latus* from China, with 91.93% similarity, but only 83.07% similarity to *K. lutjanus* when the *rnl* gene was used for sequence alignment (Figure 6C,D).

## 4. Discussion

The genus of *Kudoa* Meglitsch, 1947, currently includes more than 100 nominal species from a wide range of fish and geographical regions [24]. *Kudoa iwatai* was first described in *Pagrus major* and *Oplegnathus punctatus* from Japan [25] and has been detected in 13 different fish families spread throughout Japan, Israel, and Korea [20,22,23]. Compared with the myxosporeans, the most reported parasites with specificity to the host, *Kudoa* spp. display low host specificity [26]. It was recorded that *Kudoa thyrsites*, *Kudoa nova*, and *Kudoa iwatai* were isolated from 38, 20, and 19 hosts, respectively [27,28]. There have been only four reports of *Kudoa* infections in farmed fish in China over the past 70 years, mainly in maricultured fish in Guangdong and Fujian provinces [29,30,31,32]. Recently, Li redescribed the four *Kudoa* spp., including *K. bora* from *Osteomugil perusii*, *K. lutjanus*, *K. petala*, and *K. uncinata* from three Perciformes fishes. Additionally, three new species were identified from marine fishes, including *K. fujitai* n. sp., *K. acanthogobia* n. sp., and *K. guangdongensis* n. sp from *Acentrogobius chlorostigmatoides*, *O. perusii*, and *Konosirus punctatus* [33].

In the current research, large yellow croakers suspiciously infected with *Kudoa* were found during regular disease surveillance, and samples (LcK-2020) were used for morphological characterizations with a DIC microscope and TEM. LSU, SSU, mitochondrial *cox-1*, and *rnl* genes were used for sequencing, followed by phylogenetic analysis, and were identified as belonging to *Kudoa iwatai*. No massive mortality was recorded in *Kudoa*-occurring regions of diseased large yellow croaker farms. A large number of cysts were found on the fin bases, gills, brains, and muscles. Numerous cysts were observed in muscles with post-mortem myoliquefaction, which caused economic losses and reduced prices. The length and thickness of *Kudoa iwatai* spores were 8.41–11.26 μm and 7.37–11.05 μm, respectively—longer and thicker than other described *K. iwatai* spores. Most spores contained four equal spore valves from the apical view, each valve containing a polar capsule. Few spores with five polar capsules could be observed under light microscopy. In the first report on *K. iwatai*, most of the vesicles were dispersed in the trunk muscles and associated adipose tissue [25]. Subsequent research reported that *K. iwatai* could intrude the gills, kidneys, ovaries, and even the brains of fish, but no visible cysts were found in the internal organs of infected rock bream (*Oplegnathus fasciatus*) from South Korea [20]. Aberrant spores with five and eight polar capsules were observed from *Sparus aurate*, *Pagrus major*, and *Chaetodon paucifaatus* with *Kudoa* infection [23], but no abnormal spores were found in *K. iwatai* in *Oplegnathus fasciatus* from South Korea [20]. This current report of large yellow croaker infected with *Kudoa iwatai* in Zhejiang province could be the first report and member of the family Sciaenidae in China.

The life history of *Kudoa* is not fully understood; researchers speculate that there are two different phases: the first or actinospore phase in annelids and the second or myxosporea phase in fish. White pseudocysts are considered one of the few life cycle stages of *Kudoa* [12]. Moreover, polar capsules contain a coiled, projectile, penetrating structure called a polar filament that is released and used as an anchor to attach to the host during infection [7]. Some mature spores of *Kudoa islandica* n. sp. have been found to have protruding polar filaments [34]. In the current study, the authors observed white pseudocysts, and the protruding polar filament was measured as 9.08–12.15 μm in length. No actinospore-like parasite or annelid-like organisms were observed, possibly because large yellow croakers are mainly cultured in cages with rapid current regions. No records or descriptions of protruding polar filaments of *K. iwatai* have been reported by previous researchers. Since no obvious lesions or ulcerations were found on the skin of the fish, it could be assumed that the infection would possibly have been triggered when the gill epithelium was invaded with the actinospore-phase *Kudoa* by using the annelid as a vector with the help of a protruding polar filament.

For further identification, SSU, LSU, mitochondrial *cox-1*, and *rnl* genes from isolated *Kudoa* were used for phylogenetic analysis. From both SSU and LSU phylogenetic trees, *K. iwatai* LcK-2020 could be classified into the same cluster with high similarity and closer affinity to *K. iwatai* isolates from different fish hosts reported in China, Japan, and Korea. When mitochondrial *cox-1* and *rnl* genes were used for phylogenetic analysis, *K. iwatai* LcK-2020 could also be classified into the same cluster with a high similarity between closed isolates. For relative *Kudoa* species such as *K. Lutjanus* (*cox-1*: LC494281/*rnl*: LC494286)*, cox-1* and *rnl* genes were found to have 91.93% and 83.07% identity, respectively, indicating that mitochondrial genes could be considered as more useful genes in distinguishing closed and relative *Kudoa* species morphologically. The mitochondrial genes could be also used for analyzing genetic variations geographically and environmentally, revealing genetic relations for intraspecific and interspecific *Kudoa* from different hosts [16,35].

Compared to the hundred *Kudoa* species recognized in wild marine and cultured fish worldwide [14], the records of *Kudoa* were relatively few in China. Only four reports of *Kudoa* infection were published from 1930 to 2007, possibly because of the rare consumption of raw fish in China. In 2020, three new species and four species of genus *Kudoa*, including *Kudoa bora* and *Kudoa lutjanus*, were identified and reported by Li [33] from marine Perciformes and Mugiliformes in China, indicating the increasing prevalence of *Kudoa* infection. Most *Kudoa* spp. are generally considered relatively harmless to host fish and humans; few *Kudoa*, such as *Kudoa septempunctata*, were considered to cause foodborne illness, which was confirmed by oral administration of *Kudoa* spores to the suckle mouse at a dosage of 1 × 10^6^ spores/g [36]. The food poisoning and toxicity of *Kudoa iwatai* and other newly recognized species remain obscure [36,37]. Since large yellow croaker is the most important economic marine cultured and exported fish in China, it is necessary to establish detection methods for parasitic poisoning genes and a risk assessment system for better safety of fish products.

## 5. Conclusions

The isolated *Kudoa* sp. from large yellow croakers was differentiated and identified as *K. iwatai* based on morphological observations and molecular genetic characterization with respect to SSU, LSU, and mitochondrial DNA gene nucleotide sequences. This is the first report of *K. iwatai* isolated from cage-cultured large yellow croaker (*Larimichthys crocea*) in Zhejiang province, along the southeast coast of China.

## Figures and Tables

**Table 1:** Primers used to amplify and sequence the nucleotides of the rDNA, *cox-1*, and *rnl* genes.

Primer	Sequences (5′–3′)	Annealing Temperature	Length of Seq.	Reference
Ksp18S-F	GGATAACTGTGGTAAATCTAGAGC	64 °C	1300 bp	[19]
Ksp18S-R	GAGCAATTATTACAAGGCTCARTC			
Ksp28S-R	CAGCTCCATACAAGTTTACAC	64 °C	1500 bp	[19]
Kt28S1-F	CAAGACTACCTGCTGAAC			
Ki28S-F	CTATCAACCGGTGAGAACAT	59 °C	574 bp	[20]
Ki28S-R	ACGTCACACTACGCAGTTCATC			
Cox1-F3	ATGCTAATGATGGTATGGTTCCT	52 °C	471 bp	[21]
Cox1-R3	TCTCCTCAGGAAGTATGGCT			
RnL-F3	TTCACGTGTTCAGGTTCCTT	52 °C	384 bp	[21]
RnL-R3	ACCTTATCTTGCCGAATTCATCA			

**Table 2:** Measurements of *Kudoa* spp. mature spores.

Species	*Kudoa iwatai*	*Kudoa iwatai*	*Kudoa* *iwatai*	*Kudoa iwatai*	*Kudoa lutjanus*	*Kudoa* *bora*	*Kudoa petala*
Host	*Larimichthys crocea*	*Oplegnathus fasciatus*	*Acanthopagrus sclegelii*	*Sparus aurata*	*Acanthopagrus latus*	*Osteomugil perusii*	*Sillago sihama*
Locality	Zhejiang Province, China	Korea	Japan	Israeli	Guangdong Province, China	Guangdong Province, China	Fujian Province, China
Reference	Present study	[20]	[22]	[23]	[21]	[21]	[21]
Number of examined spores	100	30	20	30	20	20	16
Thickness	7.37–11.05 (9.38)	6.7–8.3 (7.8)	8.7–10.3 (10.1)	5.0–7.0 (6.0)	8.1–9.8 (8.9)	8.7–10.2 (9.7)	8.0–10.2 (9.2)
Sutural Thickness	8.4–11.26 (10.01)	NA	NA	NA	7.5–9.1 (8.2)	8.1–9.6 (9.2)	7.6–9.2 (8.5)
Width	6.98–9.98 (8.68)	9.8–11.8 (11.0)	NA	6.0–7.5 (6.8)	8.8–10.3 (9.5)	9.6–10.4 (10.1)	9.2–11.2 (10.4)
Length	7.03–9.59 (8.15)	6.5–8.3 (7.5)	7.3–8.9 (8.2)	5.0–7.0 (6.0)	7.3–8.4 (7.9)	8.3–9.4 (8.7)	7.2–8.0 (7.5)
Polar capsule length	2.02–5.25 (3.76)	2.3–4.3 (3.2)	3.7–5.0 (4.3)	2.5–3.5 (2.8)	3.6–4.5 (4.1)	4.5–6.4 (5.1)	4.0–5.2 (4.7)
Polar capsule width	1.22–3.79 (2.37)	1.3–2.5 (1.8)	1.5–2.1 (2.2)	1.5–2.0 (1.5)	1.4–1.7 (1.6)	1.9–2.5 (2.0)	1.3–1.8 (1.6)

All measurements are in micrometers and expressed as ranges with the mean values in parentheses. NA = not available.

## Data Availability

The sequences of small-subunit rRNA (SSU) and large-subunit rRNA (LSU) with lengths of 1321 bp (SSU), 1520 bp (LSU), and 574 bp (LSU) were uploaded to NCBI (GenBank accession numbers MW898141, MW898142, and MW898147). The *Cox-1* gene and the *rnl* gene of LcK-2020 with respective lengths of 458 bp and 384 bp were amplified and sequenced (GenBank accession numbers OK380943 and MZ042255).

## Supplementary Materials

The following supporting information can be downloaded at: https://www.mdpi.com/article/10.3390/ani12091145/s1, Figure S1: Detection of PCR products on *Kudoa*.; Figure S2: Comparison of partial multiple nucleotide sequence of *cox-1* gene of *K. iwatai* from different origin and *K. lutjanus*.
