# Supplementary material for: Morphological Characterization and Molecular Phylogenetic Analysis of Kudoa iwatai from Large Yellow Croaker (Larimichthys crocea) as a New Host in China
Source: Animals (Basel). 2022 Apr 29;12(9):1145. doi: 10.3390/ani12091145 (PMC9103762; doi:10.3390/ani12091145)
Supplement: Supplementary file 1 [file animals-12-01145-s001.zip › animals-1648113-supplementary.pdf]

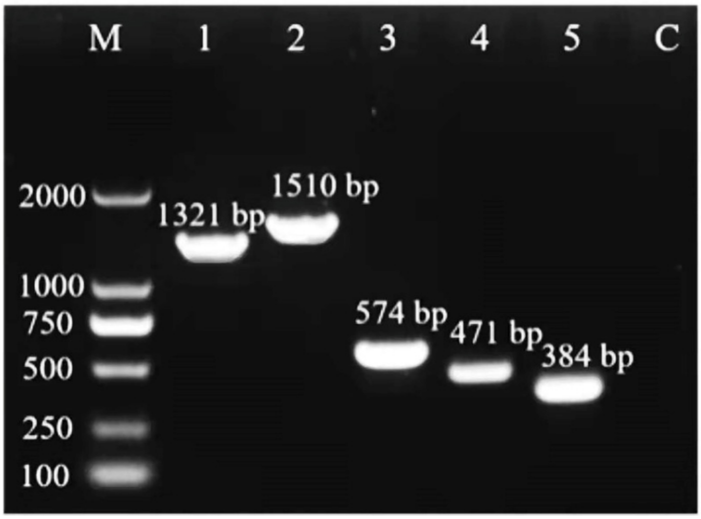

Figure S1：Detection of PCR products on *Kudoa.* Lane M: 2K DNA Marker; Lane 1, Sequence of SSU rDNA amplification (1321 bp); Lane 2: Sequence of LSU rDNA amplification (1510 bp); Lane 3: Specificity sequence of LSU rDNA of *K. iwatai* (574 bp); Lane 4: Sequence of mitochondrial of partial *cox-1* gene (471 bp); Lane 5: Sequence of mitochondrial of partial *rnl* gene (384 bp); Lane C: Control.

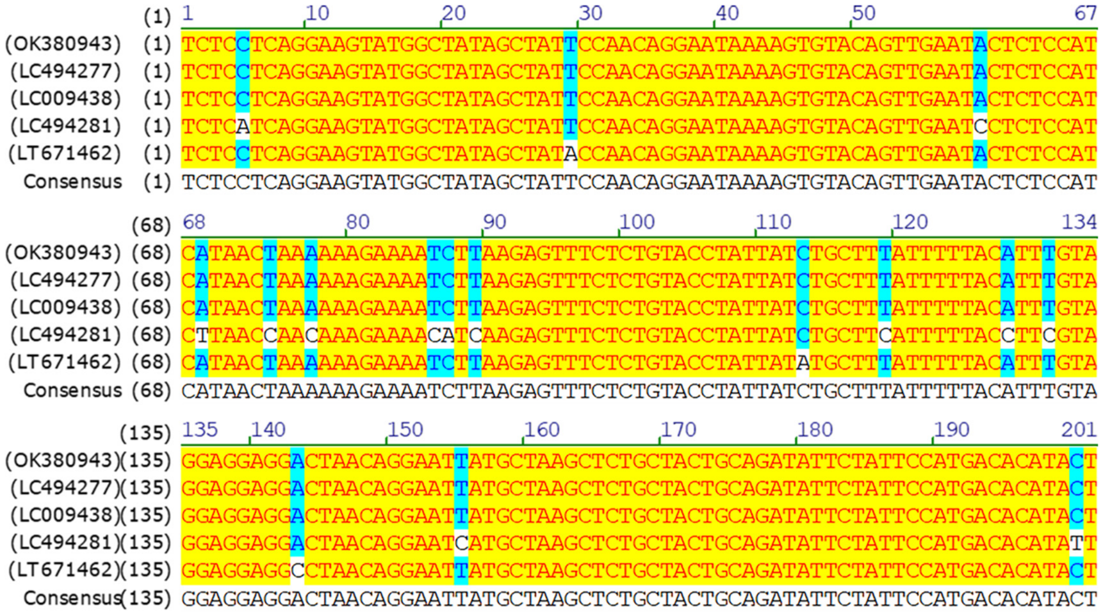

Figure S2：Comparison of partial multiple nucleotide sequence of *cox-1* gene of *K. iwatai* from different origin and *K. lutjanus*. OK380943: *K. iwatai* in *Larimichthys crocea* from China; LC494277: *K. iwatai* in *Lateolabrax japonicus* from Japan; LC009438: *K. iwatai* in *Acanthopapagrus latus* from Japan; LC494281: *K. lutjanus* in *Acanthopapagrus latus* from China; LT671462: *K. iwatai* in *Sparus aurata* from Israel. The same base pair (bp) are indicated by light yellow shading. The light blue shading indicates area with > 50% similarity.
